# Dual loss of VHL and RASSF1A enhances tumorigenicity in clear cell renal cancer

**DOI:** 10.3389/fonc.2026.1884868

**Published:** 2026-07-30

**Authors:** Howard Donninger, Rachel Ferrill, Katherine Hobbing, Michelle Vos, Geoffrey J. Clark

**Affiliations:** 1 Brown Cancer Center, Department of Medicine, University of Louisville, Louisville, KY, United States; 2 Department of Pharmacology & Toxicology, University of Louisville, Louisville, KY, United States; 3 National Institutes of Health, Bethesda, MD, United States

**Keywords:** HIF, kidney cancer, RASSF1A, transgenic, VHL

## Abstract

**Introduction:**

Clear Cell Renal cell carcinoma (ccRCC) is the most common form of kidney cancer. The VHL tumor suppressor is lost or mutated in the majority of ccRCC and hereditary mutations in VHL predispose patients to ccRCC. However, another tumor suppressor, RASSF1A is inactivated at even higher levels in ccRCC. The majority of ccRCC lose function of both proteins. This suggests there may be some functional link between them. We found that the RASSF1A and VHL proteins can form a complex, suggesting they may act in concert to suppress the tumorigenic phenotype.

**Methods:**

We generated two matched sets of Renal cancer tumor cell systems where we added back or knocked down both genes. We also made compound transgenic mice with induced, dual inactivation.

**Results:**

In the add-back system we found that the proteins cooperated to suppress 3D cell growth and sensitized cells to apoptosis inducing agents. In the knockdown system, we found the double loss cooperated to promote growth in 3D. In cell line xenografts, we found that the loss of VHL alone was insufficient to promote tumorigenesis, but it acted as an accelerant on tumor growth by RASSF1A deficient cells, which showed enhanced vascularization differential deregulation of HIF1α compared to HIF2α.

**Discussion:**

This is the first report of an interaction between RASSF1A and VHL and may explain why RASSF1A is so frequently inactivated in Renal cancer. However, further studies in transgenic mice showed that dual inactivation of the tumor suppressors did not result in the formation of tumors or macrocysts. Thus, additional genetic lesions are likely required for the development of ccRCC.

## Introduction

According to the American Cancer Society over 14,000 people die of kidney cancer per year in the USA alone. The most common form of Renal carcinoma is clear cell Renal Cell Carcinoma (ccRCC), making up around 80% of all cases (1). Although the overall 10-year survival rates are relatively good compared to other cancer types (~70%), ccRCC has the potential to metastasize, at which point it becomes much more dangerous (2).

The von Hippel-Lindau gene (VHL) is thought to be one of the most important tumor suppressors contributing to the development of ccRCC yet identified (3, 4). VHL is inactivated in more than 70% of sporadic primary ccRCC and a familial form of ccRCC is the most common manifestation of individuals carrying inheritable VHL mutations (5). VHL appears to have multiple functions, but the best characterized involves modulating the stability of the alpha subunit of the HIF transcription factors (4, 6). VHL serves as the substrate recognition component of a complex including ElonginC/B, culin2 and RBX (known as the VCB-CR complex) which acts to ubiquitinate the HIFα subunits leading to their proteasomal degradation (7, 8).

There are three members of the HIF family (9). They are hypoxic dependent transcription factor systems that involve an oxygen regulated HIFα subunit and a constitutively expressed β subunit (10, 11). HIF-1α and HIF-2α are the best characterized with HIF-3α thought to play a role as a negative regulator of the other two (12). Under normal oxygen conditions, HIF-1α and HIF-2α are hydroxylated on two proline residues by prolyl hydroxylase enzymes. This allows recognition, ubiquitination and degradation by the VHL containing VCB-CR complex. However, under hypoxia, the prolyl hydroxylation is suppressed, rendering the protein insensitive to VCB-CR and causing the stabilization of the HIF dimer (13). This can translocate to the nucleus and activate hypoxia response elements in the promoters of hypoxia dependent genes, such as VEGF. This promotes vascularization, and so HIFs can be pro-tumorigenic (14–16). If VHL function is lost, then the VCB-CR complex cannot form correctly and the HIFα sub-units remain undegraded, as if they were operating under hypoxic conditions (8, 17). However, the situation may be more complex and context dependent. HIF-1α and HIF-2α have over-lapping but non-identical functions and some studies imply a tumor suppressor role for HIF-1α (18).

RASSF1A is one of the most frequently inactivated tumor suppressors in cancer. It is usually inactivated at an epigenetic level, although occasional mutants have been identified in tumors (19, 20). It is inactivated at the highest levels in ccRCC (~90%) (19, 21). RASSF1A has no intrinsic enzymatic activity and appears to act as a scaffolding protein (19). It has multiple activities, including the regulation of microtubule dynamics (22) and DNA repair (23), although its main properties seem to involve contributing to Hippo pathway (24) and Bax driven apoptosis (25, 26). Relatively recently it was shown to bind and stabilize HIF-1α (27).

The observation that RASSF1A could bind and stabilize HIF-1α in a positive feedback loop was intriguing (27), as it suggested that RASSF1A might have tumor promoting and not just tumor suppressing properties. It also made the common dual inactivation of VHL (a HIF-1α stabilizing event) and RASSF1A (a HIFα destabilizing event) in ccRCC something of a puzzle.

During our ccRCC studies, we noticed that when we co-expressed fluorescently tagged RASSF1A and VHL in live cells we could see a clear co-localization of the proteins on the microtubules. This provoked us to determine if RASSF1A and VHL might form a tumor suppressor complex and that this might explain the frequent co-inactivation of the genes in ccRCC. We confirmed that the over-expressed proteins could interact in co-immunoprecipitations. We then generated two matched sets of ccRCC cancer cell lines where we either added back RASSF1A and VHL to negative system or knocked them out in a positive system. We then assayed the cell sets *in vitro* and *in vivo* to determine if we could see any co-operative effects. *In vitro*, we observed enhanced biological consequences in cells that had lost both tumor suppressors. *In vivo*, RASSF1A loss seemed to be the most important component of tumorigenicity but VHL loss served as an accelerant. This correlated with evidence of enhanced angiogenic activity in the double knockdown tumors. Thus, VHL loss appears to enhance the tumorigenic effects RASSF1A loss *in vivo*, explaining the dual inactivation seen so often in human tumors. The mechanism may involve enhanced angiogenesis.

However, although we could detect enhanced tumorigenesis due to VHL/RASSF1A inactivation in human tumor cells, we did not observe any tumors in a VHL/RASSF1A dual inactivation transgenic mouse system. A similar result was recently reported by Catalano et al. (28). Therefore, it appears that additional genetic lesions are required to facilitate the effects of VHL/RASSF1A loss of function on ccRCC development.

## Materials and methods

### Plasmids

GFP-VHL and HA-VHL were obtained from Addgene, RFP-RASSF1A has been described previously (23), pLRT has been described previously (29), shRASSF1A has been described previously (23), anti-VHL was pGIPZ clone V2LHS_263273 (Open Biosystems, Huntsville). HA RASSF1A constructs (wild type and mutant) have been described previously (23, 30).

### Cells and tissue culture

COS-7, 293-T and Caki-1 cells were obtained from the ATCC and used within six months. Cells were tested for mycoplasma with a MycoAlert kit (Lonza, NJ). The 786–0 VHL +/- system was a generous gift from WG Kaelin (31). Cells were cultured in Dulbecco’s Modified Eagle Medium (DMEM) supplemented with 10% fetal bovine serum (FBS) at 37 degrees in 5% CO2. pLRT vector induction was achieved by adding 100ng/ml of doxycycline to the media essentially as described in (29). Soft agar assays were performed as described previously (32). Fluorescent GFP and RFP fusion proteins were visualized at room temperature in live cells in growth medium using an Olympus IX50 inverted microscope with a UPlanFl 100×/1.3 oil immersion objective. Stable transfectants were generated by transfecting 200 ng of the appropriate plasmid using Lipofectamine 3000 according to the manufacturer’s protocol. Stable transfectants were selected in 1.5 μg/mL puromycin for the shRNA constructs and 10 ug/ml Blasticidin for the pLRT constructs. Transient transfections were performed using jetPRIME (Polyplus transfection, Illkirch, France) according to the manufacturer’s protocol and 1 ug of each plasmid.

### Apoptosis assays

The 786–0 VHL/RASSF1A +/- cell system was induced with100ng/ml Doxycycline for 48 hours prior to challenge with either 10ng/ml TNFα for 6 hours or TRAIL for 24 hours. Apoptosis was measured using the caspase-Glo kit from Promega as described in the manufacturer’s protocol.

### Antibodies

Anti-GFP was obtained from Santa Cruz Biotechnology (SC-9996), Anti-HA from Covance (MMS-101P), anti-β-actin from Sigma Aldrich (Cat# A5441) and anti-RASSF1A from Abcam (clone 3F3), anti-VHL #2738 (Cell Signaling).

### Immunoprecipitation and western blotting

Cellular lysates for immunoprecipitation were prepared using a modified RIPA buffer (50mM Tris-HCl, pH 7.4, 200mM NaCl, 1% NP-40). Precleared lysate was incubated with GFP-Trap^®^ beads (ABP-nAb-GFPA100) (Allele Biotech, BG) or with primary antibody or control IgG followed by protein A/G beads (Cat #20421) (eBioscience) and were washed with lysis buffer. Proteins were run on a 4-15% Tris-Glycine gel (Bio-Rad) and transferred to 0.2μm Nitrocellulose (Bio-Rad). Blots were developed using West Pico Enhanced ECL (Pierce Biotechnology) or West Femto Enhanced ECL (Pierce). Antibody dilutions followed the manufacturer’s recommended protocol.

### Xenograft experiments

Cells were plated at low density and harvested in log phase growth. Cells were resuspended in PBS (Phosphate Buffered Saline) at a concentration of 10 × 10^6^/mL, and 100 μL containing 1 × 10^6^ cells was injected subcutaneously into the left flank of NRG mice (Jackson Laboratories, Bar Harbor, ME, USA) (n = 6 per group). All animal experiments were approved by the University of Louisville IACUC committee (protocol 18236).

### Immunohistochemistry

Frozen tumor sections were thawed, fixed for 5 minutes with 2% buffered formalin, washed 3 times with 0.05M Tris HCL with 1% triton X, and blocked 20 minutes in 10% heat inactivated normal goat serum. Slides were incubated in either HIF2α, HIF-1α (Thermo Fisher, Rockford, IL) or VEGF (Oncogene, Pittsfield, MA) 1:250 overnight. Slides were then incubated in goat anti-rabbit Alexaflor 488 (Invitrogen Molecular Probes, Eugene, OR) 1:500–1 h room temperature, washed in Tris HCl, and examined for green fluorescence. Slides were counted for positive cells per 3 representative fields.

### Transgenic mice

PEPCK-cre VHL mice (33) were a generous gift from Dr. Volker Haase (Vanderbilt university). RASSF1A knockout mice (34) were a generous gift of Dr. Gerd Pfeiffer (Van Andel Institute). Animals were crossbred and genotyped as described in (33, 34). Kidney recombination at the VHL locus was confirmed by PCR, essentially as described in (33). At 12 months of age, approximately equal numbers of male and female mice were sacrificed for examination. Kidney sections were stained with H and E and examined by a board-certified pathologist under blinded conditions. Experimental cre groups were 4 x(1A+/+, VHL -/-), 7x (1A-/+/VHL-/-), 13x (1A -/+, VHL -/+) and 4x (1A+/+, VHL +/+).

### Statistical analysis

GraphPad Prism version 6.0 (GraphPad Software Inc., La Jolla, CA) was used to evaluate statistical significance by One way Anova and Student’s *t* tests.

## Results

### RASSF1A and VHL can form a complex

**Figure 1:**
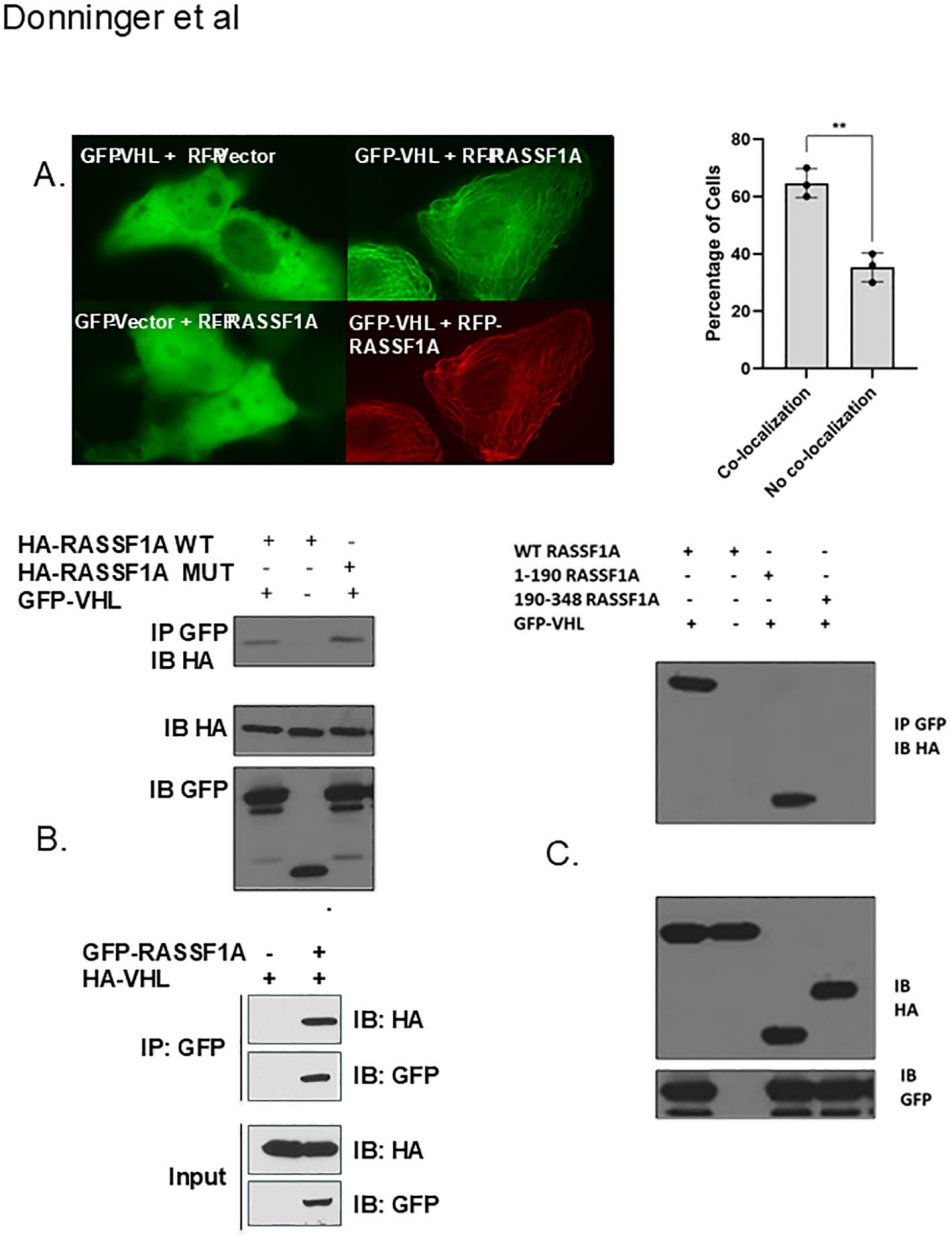
VHL and RASSF1A can form a complex. **(A)** Co-localization of VHL and RASSF1A in live cells. Left panels: GFP-VHL is cytoplasmic (top left) but when co-transfected into COS-7 cells with RFP-RASSF1A it re-localizes to microtubules with the RASSF1A (right panel). GFP vector and RFP-RASSF1A alone are shown in the control panels below. Right panel: Quantification of three separate experiments for co-localization. 100 dual positive cells were counted for each point. ** p = <.01. **(B)** Co-immunoprecipitation of RASSF1A and VHL in a complex. (Top panels) GFP-VHL and HA-RASSF1A (wild type and a common RASSF1A point mutant (K21Q)) were co-transfected into HEK293-T cells. After 24 hours, the cells were lysed and the GFP RASSF1A immunoprecipitated (IP). The immunoprecipitate was then immunoblotted (IB) for GFP and HA. The lower two panels show the levels of GFP-RASSF1A and HA-VHL in the input lysate. The reciprocal experiment was performed with GFP-RASSF1A and HA-VHL (bottom panels). **(C)** Deletion mapping of the site of interaction on RASSF1A. GFP-VHL and HA-RASSF1A (wild type and deletion mutants) were cotransfected into HEK293-T cells. After 24 hours, the cells were lysed and the GFP-VHL immunoprecipitated (IP). The immunoprecipitate was then immunoblotted (IB) HA. The lower two panels show the levels of GFP-RASSF1A and HA-VHL proteins in the input lysate.

RASSF1A is partially localized to the microtubules and can act to stabilize them (22). When we co-expressed GFP-tagged VHL and RFP tagged RASSF1A in COS-7 cells in transient transfections, we observed clear co-localization of RASSF1A and VHL on the microtubules (**Figure 1A**). This could be observed in the majority, but not all, of the transfected cells (**Figure 1A**, right panel). To confirm that this is a stable interaction, we transiently co-transfected epitope tagged expression vectors of RASSF1A and VHL into HEK293T cells and performed reciprocal co-immunoprecipitations (**Figure 1B** upper and lower panels). We also included a common tumor derived point mutation of RASSF1A (K21Q). Both wild type and mutant forms of RASSF1 co-immunoprecipitated with VHL. To map the site of the interaction, we co-transfected RASSF1A deletion mutations and VHL into HEK-293T cells. We then performed co-immune precipitations (IP) and examined the degree of stable interaction of VHL and RASSF1A by immunoblot of the precipitate (IB). We found we could detect a stable complex between the full length proteins and the interaction mapped to the N-terminal 190 aa (**Figure 1C**). The 190–340 amino acid fragment did not appear to bind.

### RASSF1A induction co-operates with VHL to suppress 3D growth in soft agar

**Figure 2:**
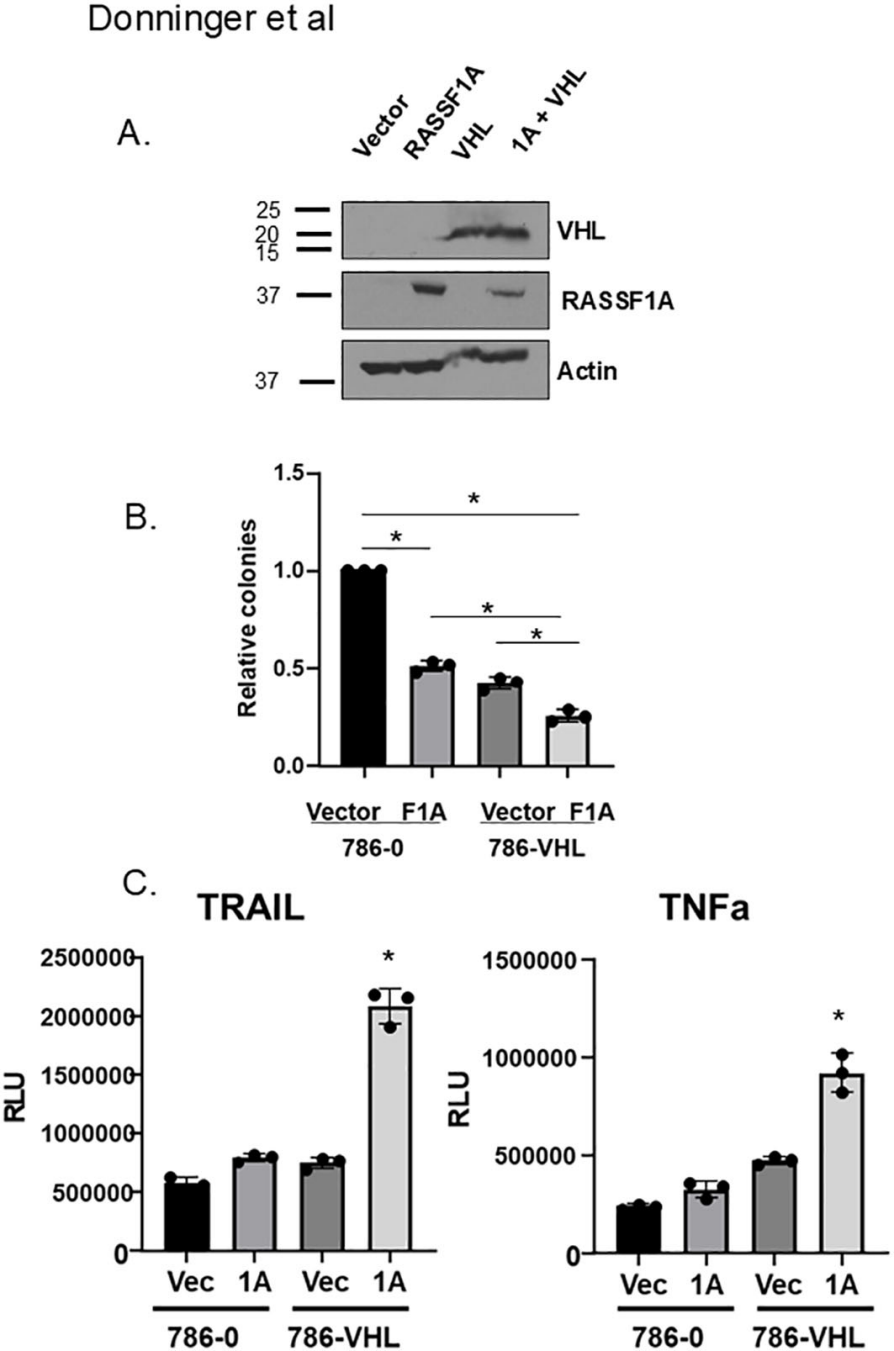
RASSF1A and VHL co-operate to suppress growth in 3D and sensitize 786–0 ccRCC cells to apoptosis. **(A)** Expression of VHL and RASSF1A in the matched set. The 786–0 ccRCC cell line +/- VHL system was stably transfected with an inducible RASSF1A construct, induced and immunoblotted for RASSF1A after 48 hours. Actin serves as a loading control below. **(B)** Soft agar colony formation of the 786–0 matched set. The VHL+/-, RASSF1A +/- matched cell set was plated in soft agar in the presence of doxycycline. Colonies were scored after three weeks. Data is presented relative to the vector/vector (VHL-/RASSF1A-) cell line. The RASSF1A+/VHL+ line showed a statistically significant decrease in relative colony formation compared to the vector and cell systems positive for only one of the tumor suppressors (p = .0004). vv/v/F1A: p = .0001, F1A/VHL vs F1A: p = .026, F1A vs F1A/VHL: p = .004. n = 3. Anova p = .0001. **(C)** TNFα and TRAIL response of the matched set. The 786–0 VHL/RASSF1A +/- cell system was induced with Doxycycline for 48 hours prior to challenge with (left panel), 10ng/ml TNFα for 6 hours, (right panel) TRAIL for 24 hours Apoptosis was measured using the caspase-Glo kit from Promega. The restoration of RASSF1A and VHL enhances the TRAIL response relative to the vector control (p = .001, n =3) and to TNFα (n =3, p =.003). *Denotes p value less than.05.

To examine the effects of RASSF1A expression in the presence or absence of VHL, we used an inducible RASSF1A expression construct and a previously established VHL+/- cell system. 786–0 cells are a RASSF1A negative, VHL negative renal carcinoma cell line (31, 35). A matched pair has been generated where VHL has been restored to one set (31). We stably transfected these cells with either pLRT vector or pLRT-RASSF1A, a doxycycline inducible expression vector (29). We then confirmed RASSF1A could be expressed when induced in the matched set (**Figure 2A**). We then plated the matched set in soft agar, a 3D growth model that is considered one of the best predictors of *in vivo* tumor growth (36). We found that the presence of both tumor suppressors gave a statistically significant increase in the suppression of 3D growth (**Figure 2B**).

RASSF1A is a component of both the TNFα and TRAIL apoptotic response pathways (20, 37, 38). When we induced RASSF1A in the cell system and challenged with TRAIL or TNFα, in both cases the cells gave a sharply enhanced and statistically significant caspase activation response when both VHL and RASSF1A proteins were both present, as measured by the caspase-Glo assay system (**Figure 2C**). Although RASSF1 alterations alone had little effect.

### Dual suppression of RASSF1A and VHL enhances the growth in soft agar of ccRCC cells

**Figure 3:**
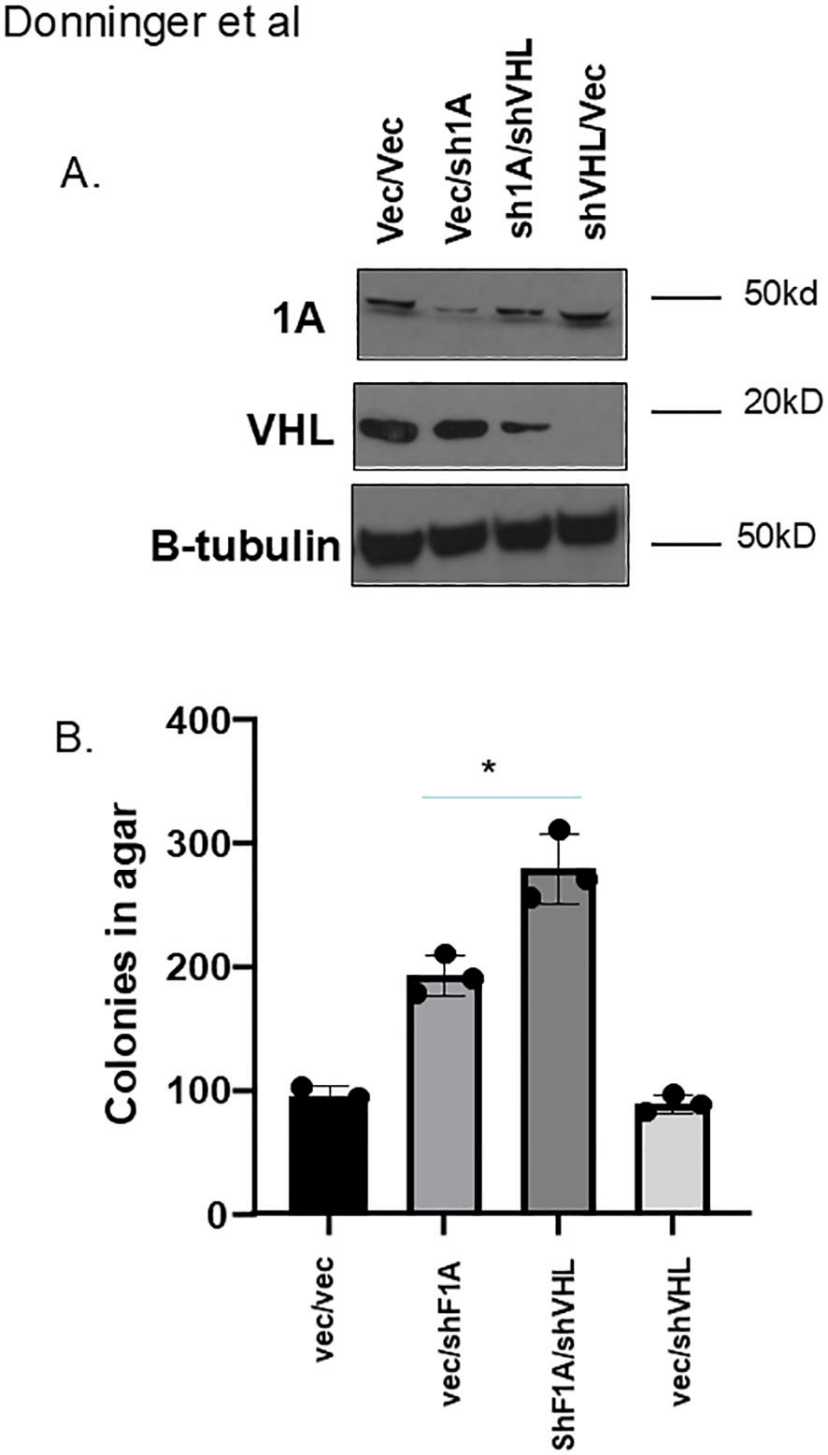
RASSF1A and VHL co-operate to suppress growth in 3D in Caki-1 ccRCC cells. **(A)** Expression of VHL and RASSF1A in the matched set. Caki-1 cells were sequentially stably selected with shRNA constructs against VHL and RASSF1A. The resultant cells were analyzed by Immuno-blot to confirm target gene knockdown. B-tubulin served as a loading control. **(B)** Soft agar assays of the matched set. The matched knockdown cell system was assayed for growth in 3D, an *in vitro* predictor of tumorigenicity. N = 3. The RASSF1A knockdown cells gave more colonies than the vector control (p = .008) as did the dual knockdown (p = .0004). The dual knockdown exhibits greater colony formation that the RASSF1A knockdown (p = 0105). * Denotes p value less than.05.

The Caki-1 cell line is positive for both VHL (39) and RASSF1A expression (35). We used sequential stable transfection of shRNA constructs into the cells to generate a matched set of cell lines that were knocked down singly or dually for the proteins. Early passage, pooled populations were used for subsequent experiments. We validated the knockdown by Western analysis (**Figure 3A**) and then assayed the effects of the knockdown in 3D soft agar assays (**Figure 3B**). We found that the RASSF1A knockdown cells exhibited a significant increase in tumor colony growth. The double knockdown cells exhibited a further increase.

### VHL loss accelerates tumor formation due to RASSF1A loss

Both VHL and RASSF1A can act on the HIF transcription factor α subunits, which are heavily influenced by hypoxia (17, 27). These factors can promote vascularization of the tumor (40), which is difficult to model *in vitro*. In order to examine the full effects of the tumor suppressors on tumor growth, we performed xenograft assays with the Caki-1 knockdown matched set. NRG immune-compromised mice were injected in the flank with the 4 matched sets of cell lines and tumor growth monitored once per week. We found that at six weeks, all the mice injected with the double knockdown cells had developed tumors, but no tumors were detectable in the other groups. We extended the experiment and observed that after a further 4 weeks we could detect a tumor in one of the control mice and all RASSF1A knockdown mice, but still none of the VHL knockdown mice had detectable tumors (**Table 1**).

**Table 1:** Effects on tumorigenicity of suppressing RASSF1A and VHL expression in xenografts.

Time	Vec/Vec	Vec/shVHL	shF1A/Vec	shF1A/shVHL
6 weeks	0	0	0	6
10 weeks	1	0	6	6

**Figure 4:**
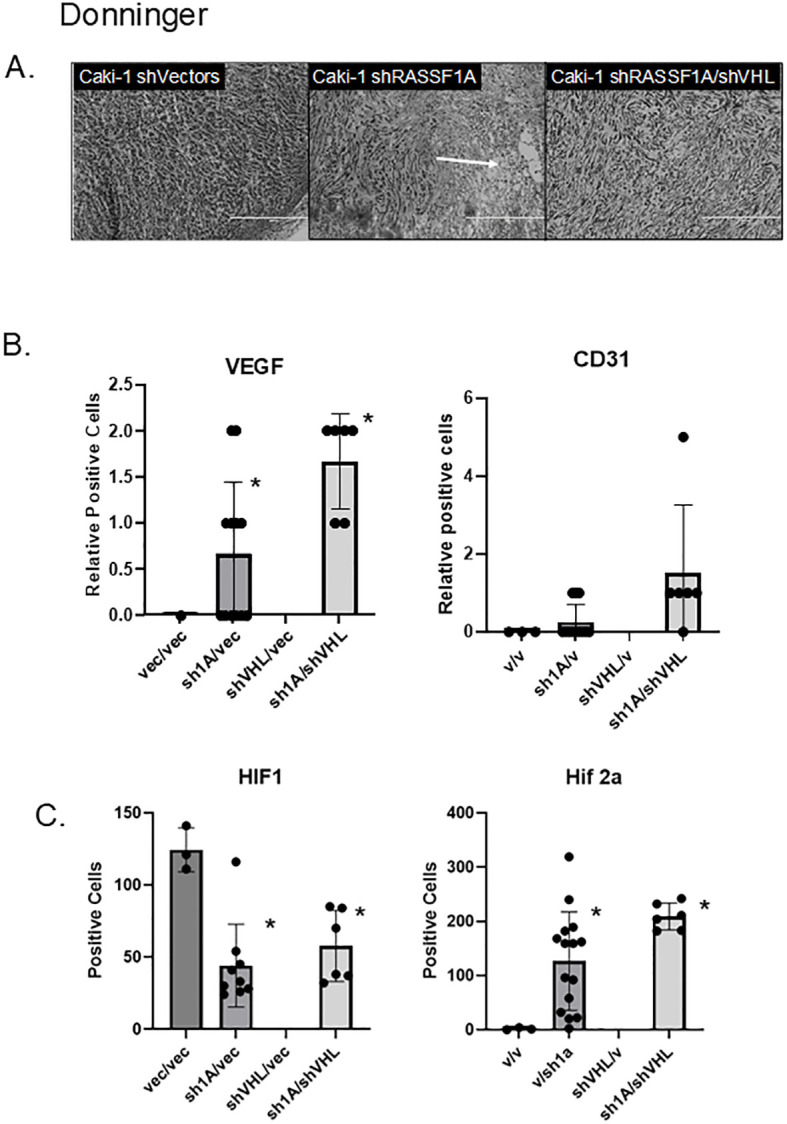
Characterization of Caki-1 tumors knocked down for RASSF1A and/or VHL. **(A)** Representative picture of H&E stained slides from the tumors. Note, no tumors arose in the VHL knockdown tumor animals. White arrow illustrates an area of necrosis. Each dot is one slide count. **(B)** i. VEGF expression: Immunohistochemistry quantification of sections from the tumors showed more cells were positive for VEGF in the knockdown tumors (Anova, p = .007), and that the double knockdown was significantly more positive than the single RASSF1A knockdown samples (p=.003). No tumors arose in the VHL knockdown alone. ii. CD31 expression: Similar experiments were performed using CD31 as a marker. Here a trend was observed but it was not significant. P = .065. **(C)** HIF expression: HIF- α1 (left panel), IHC showed that the knockdown tumors, both single and double showed less HIF-1α expression than the vector samples (p = .004). HIF-2α (right panel), IHC showed that the knockdown tumors, both single and double showed more HIF-2α expression than the vector samples (p = .031). The double knockdown showed the strongest staining but variability in the single knockdown slides prevents this from being statistically more significant.

We took sections from the tumors and stained them with H & E. The tumors from the mice injected with the RASSF1A knockdown cells appeared to show considerable necrosis that was not apparent in the tumors from the double knockdown cells or in the small, slow growing control tumor (**Figure 4A**). The necrosis could be a marker of insufficient vascularization. We then performed immunohistochemistry to quantify the level of HIF expression and for markers of vascularization. We observed an elevated level of VEGF, an inducer of vascularization (41) in the double knockdown tumors that did not exhibit evidence of necrosis (**Figure 4B**, left panel). We also examined levels of CD31 as a further marker of angiogenesis (42). Although this marker showed a trend towards elevation in the double knockdown samples, it was not statistically significant (**Figure 4B**, right panel). Finally, we then examined HIF levels in the tumor sections. Whereas HIF1-α was lower than the vector control, HIF2-α was elevated (**Figure 4C**).

### Dual inactivation of VHL and RASSF1A in the kidney does not induce ccRCC

**Figure 5:**
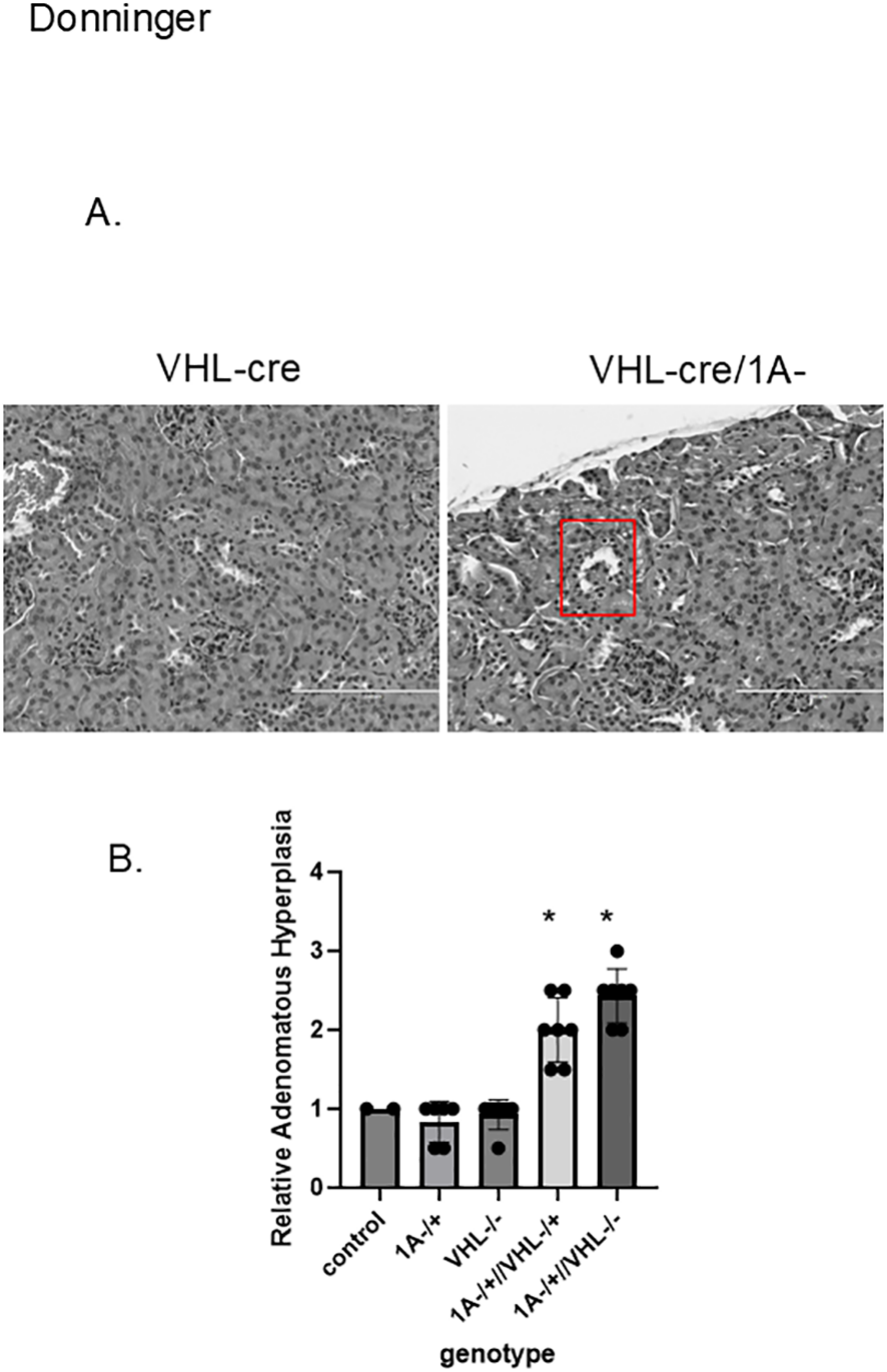
Histopathological examination of kidneys from transgenic mice. **(A)** Representative H & E section of kidney from VHL-cre and dual VHL/RASSF1A inactivation mouse. Sections show no macro-cysts or tumors, but highlights an area of Adenomatous hyperplasia (red box) in the VHL/RASSF1A compound transgenic mouse kidney. **(B)** Quantification of relative degree of adenomatous hyperplasia in examined kidneys. Anova gives p = .0001, *ad hoc* t-tests comparing 1A hets-/+/VHL-/+ and 1A/Het/VHL-/- to control give p values of.0131 and.0008 respectively. Each dot is one slide scored, * = p <.05.

To definitively determine the function of dual VHL/RASSF1A inactivation in kidney cancer we generated compound transgenic mice with heterozygous RASSF1A and single or dual knockout of VHL in the kidneys. After one year we sacrificed the animals and examined the kidneys for the presence of large cysts or tumors. We observed no such formation. Recently, similar experiments have come to the same conclusion (28). Close examination did suggest a modest but significant increase in adenomatous hyperplasia in dual knockout animals, but no evidence of malignant disease (**Figure 5**).

## Discussion

The most common form of kidney cancer is ccRCC. The 10-year survival rate is relatively good compared to some cancers, but this is largely because surgery before the tumor metastasizes can be very effective. Once metastasis has occurred, the prospects become much worse and despite some recent advances, therapeutic options remain poorly effective (43, 44).

VHL is the most commonly inactivated tumor suppressor in ccRCC and humans who have an inheritable mutation in VHL have an enhanced propensity to develop ccRCC (4). However, this can take some time and experimentally VHL inactivation is insufficient to promote the tumorigenic phenotype on its own (4). The additional genetic components that contribute to ccRCC development with VHL are not fully understood.

VHL has been ascribed multiple functions, but the best characterized and perhaps most important is the regulation of the HIF transcription factors (17, 45). Under normoxic conditions a VHL multi-protein complex (VCR-CR) modifies the α sub-units of HIF with ubiquitin, targeting them for degradation by the proteosome. Hypoxia, or loss of expression or mutations in VHL that disrupt the VCR-CR complex render the complex unable to modulate HIF (46–48). This results in aberrant activation of hypoxic response elements in genes regulated by the HIF-1α and HIF-2α transcription factor sub-units. Many of these genes play an important role in tumorigenesis, particularly in mediating the neo-vascularization required for optimal tumor growth (49, 50).

RASSF1A is conventionally considered to be a tumor suppressor. It is noticeable that of all the cancers in which it is inactivated, ccRCC exhibits the highest frequency (19). RASSF1A is a scaffolding protein and has a well-characterized role in Hippo pathway and Bax pathway mediated apoptosis (19, 20). However, it can also modulate a range of other biological processes including DNA repair and microtubule dynamics. Despite its tumor suppressing capabilities, RASSF1A has been shown to bind and stabilize HIF-1α (27). Indeed, there appears to be something of a feed-forward loop as HIF-1α can active transcription from the RASSF1A promoter, so RASSF1A levels increase under hypoxia. In this particular context, RASSF1A may have tumor promoting activities. As RASSF1A and VHL are often both impaired in ccRCC and as VHL has been reported to modulate microtubule dynamics (51), like RASSF1A (22), we wondered if there might be an interaction between the proteins. We were able to see clear complex formation with over-expression studies, although technical limitations of the RASSF1A antibodies made confirming the interaction at an endogenous level difficult.

To examine the biological interaction, we used two different ccRCC cell systems, an “add back” and a knock-down system to investigate the biological consequences of the dual inactivation so often seen in primary renal tumors. In the add back system 786–0 cell system, there was a statistically significant enhanced effect on suppressing the ability to grow in soft agar when both RASSF1A and VHL were present. In the dual knockdown system (Caki-1 cells) loss of RASSF1A enhanced colony formation and this was further increased, modestly, when both genes were knocked down. Overall, the effects *in vitro* of dual inactivation were not dramatic. However, we did measure a marked increased sensitivity to apoptotic stimuli (TRAIL and TNFα) in the double positive add-back cells. TNFα has been shown to serve as pro-growth factor that is secreted in an autocrine fashion in ccRCC (52). Here we see it inducing apoptosis when both RASSF1A and VHL are present, but not RASSF1A alone. This is a little unexpected, but RASSF1A appears to interact with p53 at biologically several levels (53) and although both cell lines used are technically p53 wild type, they have both been reported to have deficiencies in p53 signaling and function (54). This may restrict the apoptotic potential of RASSF1A alone.

The HIF transcription factors play a critical role in ccRCC development (17). So much so that targeted anti-HIF therapeutics have been developed, although not yet approved (55). The majority of ccRCC lose expression of both VHL and RASSF1A. Yet VHL and RASSF1A have a potentially antagonistic effect on HIF-1α stability (13, 27). Moreover, we found they can form a complex and thus, may influence each other’s activity. So, the role of the dual inactivation of VHL and RASSF1A on HIF regulation in ccRCC is unclear. *In vitro* studies gave us conflicting results that were frustratingly variable, possibly due to the confounding effects of trying to use artificial modes of hypoxia. So, to try and answer the questions of the role of dual inactivation in actual tumor growth and the effects on HIFs, we performed *in vivo* xenograft assays. Here we anticipated a true hypoxic tumor microenvironment.

The Caki-1 cell line we used retains VHL (39) and RASSF1A expression (35). It is poorly tumorigenic on its own and we only obtained 1/6 mice that gave a tumor after 10 weeks post injection from the control, vector transfected cells. Mice injected with cells knocked down for VHL alone were even worse, and no tumors were obtained from this group at all. Knocking down RASSF1A alone resulted in tumors in all the injected mice, but they arose more slowly than in mice injected with the double knockdown cells. This suggests that it is the RASSF1A that is the most important genetic event driving the tumor, but that VHL loss can accelerate the tumor development. It is to be noted; these experiments were performed in immune-compromised mice. Therefore, any pro-tumorigenic effects of VHL loss on the tumor immune-microenvironment will be absent (56).

We used histopathology and immunohistochemistry to characterize the tumors. It was quite obvious that the slower growing RASSF1A alone knockdown tumors had areas of necrosis suggestive of poor vascularization compared to the double knockdown tumors. We saw no such effects in the tumor arising from the vector cells, but this was much smaller and might not be large enough to suffer insufficient vascularization. When we performed immunohistochemistry we saw enhanced levels of VEGF, a key pro-vascularization factor that is induced by HIF transcription factors in the double knockdown tumors. When we then examined the tumors for HIF-1α expression we noticed that RASSF1A negative tumors all exhibited less HIF-1α than the vector control tumor. This fits with the observations of Dabral et al. (27) that RASSF1A is required to stabilize HIF-1α. However, Dabral et al. did not report on the effects on HIF-2α. In the RASSF1A negative tumors we saw upregulated HIF-2α compared to the vector and this was further upregulated in the double knockdown tumors. Thus, we see evidence of enhanced vascularization and HIF-2α but not HIF-1α activity in the double knockdown cells.

HIF-1α and HIF-2α have overlapping but non-identical functions (18, 57). Some genetic data suggests that at least in ccRCC, HIF-1α may play a tumor suppressor role while it is HIF-2α that is most important for tumor progression (58). However, other studies have confirmed an essential role in tumor initiation for HIF-1α (59). The importance of each HIF to ccRCC may be influenced by the presence or absence of other factors, such RASSF1A. Perhaps this may explain why RASSF1A and VHL are both inactivated at the same time in so many ccRCC tumors.

Recently, a compound transgenic mouse system has been generated with VHL and RASSF1A knockout in the kidney (28). The authors report some effects on gene transcription but no cyst or tumor development. We had performed similar studies and observed similar null results. We did observe a tendency towards enhanced Adenomatous hyperplasia in the dual inactivated animals after one year, but this was quite subtle. Thus, it would appear that additional genetic lesions are required to co-operate with RASSF1A and VHL deficiency to promote kidney cancer.

## Conclusion

RASSF1A and VHL can form a protein complex in cells. However, although inactivation of both tumor suppressors is common in ccRCC, the experimental effects of dual inactivation are modest. Thus, additional genetic lesions must be required to promote ccRCC.

## Data Availability

The raw data supporting the conclusions of this article will be made available by the authors, without undue reservation.
